# A pediatric case series of catastrophic gastrointestinal complications of posttransplant lymphoproliferative disease with increasing incidence, high association with coronavirus disease 2019, higher mortality, and a plea for early endoscopy to prevent late fatal outcome

**DOI:** 10.1186/s13256-023-04123-5

**Published:** 2023-09-19

**Authors:** Alireza Keshtkar, Fereshteh Karbasian, Hamid Reihani, Farnaz Atighi, Seyyed-Bozorgmehr Hedayati, Maryam Ataollahi, Bita Geramizadeh, Seyed Mohsen Dehghani

**Affiliations:** 1 Student Research Committee, School of Medicine, Shiraz University of Medical Sciences, Shiraz, Iran; 2 Pediatric Gastroenterology, Hepatology and Nutrition, Ali-Asghar Children’s Hospital, Iran University of Medical Sciences, Tehran, Iran; 3 Hematology Research Center, Shiraz University of Medical Sciences, Shiraz, Iran; 4 Department of Pediatric Gastroenterology, Gastroenterohepatology Research Center, Shiraz University of Medical Sciences, Shiraz, Iran; 5 Shiraz Transplant Research Center (STRC), Shiraz University of Medical Sciences, Shiraz, Iran; 6 Department of Pathology, Shiraz University of Medical Sciences, Shiraz, Iran

**Keywords:** Posttransplant lymphoproliferative disorder (PTLD), Endoscopy, Colonoscopy, COVID-19, Case series, Gastrointestinal perforation, Bleeding, Pediatric, Liver transplantation, Fatal

## Abstract

**Background:**

Posttransplant lymphoproliferative disorder is one of the most severe complications after transplantation, caused by uncontrolled proliferation of Epstein–Barr virus-positive B-cells in the setting of chronic immunosuppression. As one of the biggest transplant centers worldwide, we observed a potential increase in the number of patients with posttransplant lymphoproliferative disorder presenting with gastrointestinal symptoms in 1 year, during the coronavirus disease 2019 pandemic. There is limited information about dysregulation of the immune system following coronavirus disease 2019 infection, which may lead to Epstein–Barr virus reactivation in Epstein–Barr virus-positive B-cells and development of posttransplant lymphoproliferative disorder. Furthermore, there is no consensus in literature on a modality that can help in early diagnosis of posttransplant lymphoproliferative disorder with nonspecific gastrointestinal presentations before late and fatal complications occur.

**Case presentation:**

Our case series includes five Iranian (Persian) patients, three female (2, 2.5, and 5 years old) and two male (2 and 2.5 years old), who developed gastrointestinal posttransplant lymphoproliferative disorder after liver transplantation. All of our patients were on a similar immunosuppressant regimen and had similar Epstein–Barr virus serologic status (seronegative at time of transplantation but seropositive at time of posttransplant lymphoproliferative disorder diagnosis). Four patients had either a positive coronavirus disease 2019 polymerase chain reaction test or exposure within the family. Although all of our patients presented with nonspecific gastrointestinal symptoms, four patients developed late posttransplant lymphoproliferative disorder complications such as bowel perforation and obstruction. All five patients with gastrointestinal posttransplant lymphoproliferative disorder received chemotherapy, but only two survived and currently are continuing the therapy. In one of the surviving patients, prompt endoscopic investigation resulted in early diagnosis of posttransplant lymphoproliferative disorder and a better outcome.

**Conclusion:**

Since 80% of our patients had exposure to coronavirus, a potential relationship might be suggested between the two. Furthermore, as we witnessed in one case, urgent endoscopic investigation in immunocompromised patients presenting with gastrointestinal symptoms can improve the clinical outcomes and therefore should be considered for early diagnosis of posttransplant lymphoproliferative disorder.

**Supplementary Information:**

The online version contains supplementary material available at 10.1186/s13256-023-04123-5.

## Background

Posttransplant lymphoproliferative disorders (PTLDs) are groups of lymphoid or plasmacytic disorders that the World Health Organization (WHO) categorizes into six classes: (1) plasmacytic hyperplasia PTLD, (2) infectious mononucleosis PTLD, (3) florid follicular hyperplasia PTLD, (4) polymorphic PTLD, (5) monomorphic PTLD (B- and T-/NK-cell types), and (6) classical Hodgkin’s lymphoma PTLD [1].

PTLD is a severe complication in patients who undergo transplantation surgery and receive subsequent immunosuppressive therapy. Its incidence varies in different solid organ transplantations, which might be due to the severity of immunosuppressive therapy and the quantity of transplanted lymphoid tissue [2]. To the best of our knowledge, Starz *et al.* were the first researchers to use the term “PTLD” [3].

There are several risk factors for PTLD, including immunosuppression duration and its degree, infections especially by Epstein–Barr virus (EBV), race, recipient age, allograft type, and genetic factors [4]. Liver transplantation is one of the most common transplantation surgeries in pediatrics, and the incidence of PTLD after it is approximately 5% [5, 6].

PTLD have early and late clinical manifestations. Fever, diarrhea, and abdominal pain are common early presentations. Gastrointestinal (GI) bleeding or perforation are late signs of PTLD [7, 8]. We had two patients who presented with perforation, a very rare complication in PTLD, one of whom experienced recurrent perforation.

The incidence rate of early gastrointestinal (GI) manifestations as the first symptoms of PTLD after liver transplantation varies in different studies. For instance, in an original study performed by Barış *et al.*, it was reported that, out of 236 patients who underwent liver transplantation over an 18-year period, eight patients were diagnosed with PTLD, and only 2 of them presented with GI manifestations. Similarly, in a 13-year cross-sectional study conducted by Eshraghian *et al.* at our institute, out of 40 patients with PTLD, 15 presented with abdominal pain and 5 with diarrhea [9, 10]. In this case series, we observed an apparent increase in the number of PTLD cases presented with GI manifestations, with five such cases occurring within 1 year.

## Case presentation

**Fig. 1:**
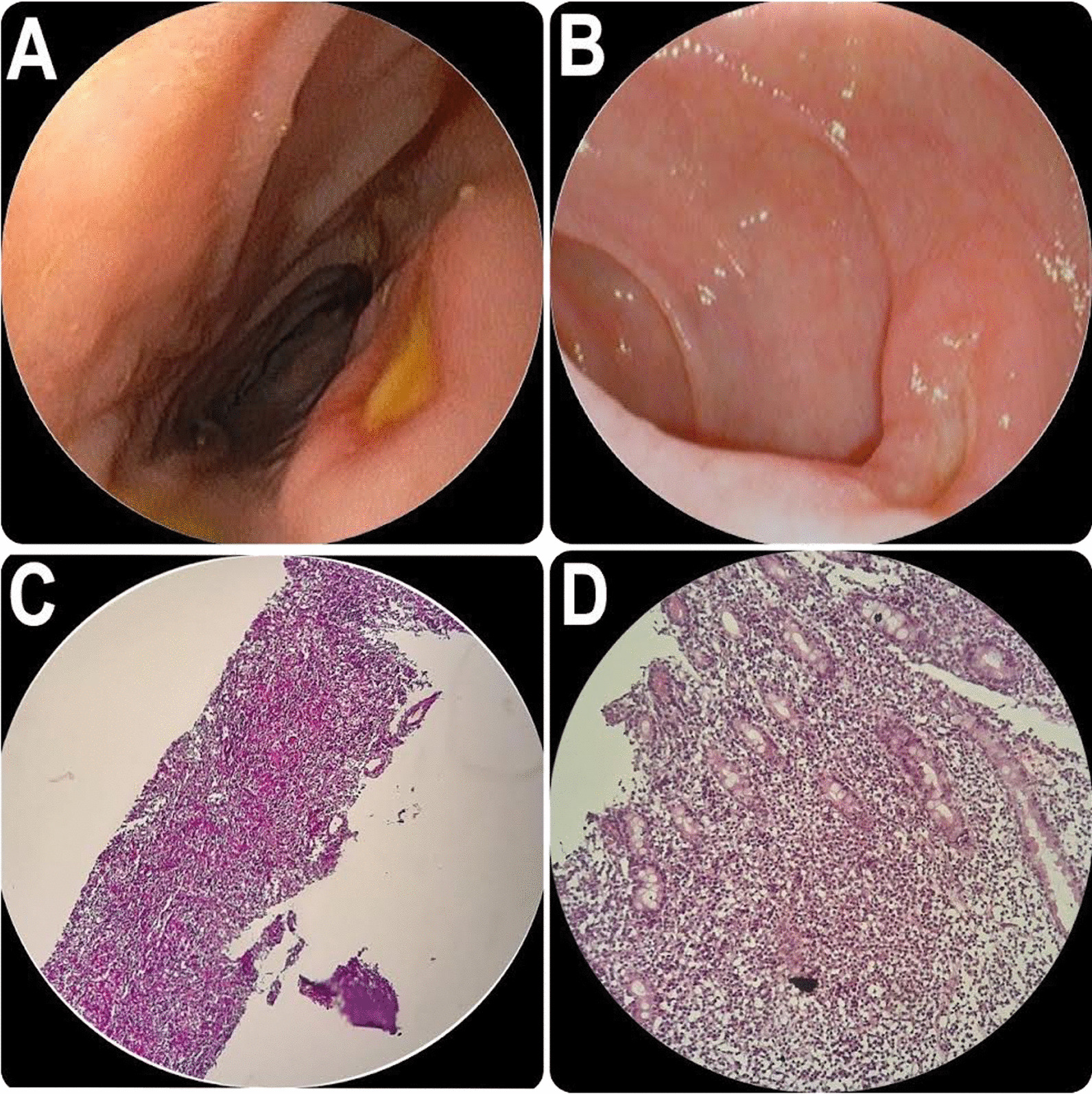
**A** Case 4 colonoscopy picture. **B** Case 3 endoscopy picture. **C** Lymph node biopsy of case 2 shows diffuse infiltration of lymphoma cells which are all positive for CD20. **D** Small intestine biopsy of case 2 shows diffuse infiltration of atypical lymphocytes which are all CD20 positive

This case presentation includes five cases of PTLD after liver transplantation with fever, diarrhea, and abdominal pain at time of admission. Significant demographic, clinical, and laboratory data are presented in Table 1. Also, colonoscopy images of case 4, endoscopy images of case 3, and pathological images of case 2 are presented in Fig. 1. The parents of all five patients gave written consent for the publication of this case series.

**Table 1:** Characteristics of our patients

Characteristic		Case number				
		Case 1	Case 2	Case 3	Case 4	Case 5
Age (years)/gender		2.5/female	2.5/female	2/male	3.5/male	5/female
Liver transplant cause		Biliary atresia	Biliary atresia	Biliary atresia	PFIC	Crigler–Najjar
Donor		Living (mother)	Living (mother)	Living (father)	Living (mother)	Living
PTLD manifestation		Fever, diarrhea	Abdominal pain, fever, diarrhea	Fever, diarrhea	Fever, diarrhea, abdominal pain	Abdominal pain, diarrhea
EBV PCR	Transplant admission	Negative	Negative	Negative	Negative	Negative
	PTLD admission	Positive	Positive	Positive	Positive	Positive
CMV PCR	Transplant admission	Negative	Negative	Positive	Negative	Negative
	PTLD admission	Negative	Negative	Positive	Negative	Positive
Tacrolimus level (ng/mL)		11.5	17.5	8.7	9.24	20
PTLD type		Monomorphic (B cell type)	Monomorphic (B cell type)	Monomorphic (B cell type)	Monomorphic (B cell type)	Monomorphic (B cell type)
Immunosuppressant regimen		Tacrolimus, prednisolone	Tacrolimus, prednisolone	Tacrolimus, prednisolone	Tacrolimus, CellCept prednisolone, everolimus	Tacrolimus, CellCept, prednisolone
Chemotherapy		Cyclophosphamide vincristine, doxorubicin, rituximab, prednisolone	Cyclophosphamide vincristine, doxorubicin, rituximab, prednisolone	Cyclophosphamide vincristine, doxorubicin, rituximab, prednisolone	Cyclophosphamide vincristine, doxorubicin, rituximab, prednisolone	Cyclophosphamide vincristine, doxorubicin, rituximab, prednisolone
F/U and outcomes		Expired	Expired	PTLD chemotherapy	Expired	PTLD chemotherapy

*PTL* posttransplant lymphoproliferative disease, *EBV* Epstein–Barr virus, *CMV* cytomegalovirus, *PFIC* progressive familial intrahepatic cholestasis

### Case 1

A 2.5-year-old Iranian (Persian) girl was referred to our hospital owing to complaints of abdominal pain and mild fever. She had history of liver transplantation due to biliary atresia when she was 7 months old without any family history for malignancy. On physical examination, abdominal guarding was observed, and her laboratory test results indicated leukopenia and a positive COVID-19 test. Sonography revealed large amounts of free fluid in the abdominal cavity, and an abdominal X-ray showed air under the diaphragm, in favor of bowel perforation. Consequently, she underwent exploratory abdominal laparotomy, during which 1 l of ascites fluid and bile, along with multiple perforations in the small bowel at the site of Roux-en-Y anastomosis, were discovered. Small bowel biopsy and immunohistochemistry (IHC) confirmed PTLD (IHC positive for LCA, PAX-5, and BCL-2). After a while, she presented with the same symptoms and was diagnosed with another bowel perforation. Her condition became complicated when the abdominal fluid culture test revealed the presence of *Acinetobacter* species. She then developed acute kidney injury and exhibited unstable vital signs, possibly indicating septic shock. Despite her critical condition and conflicting opinions regarding the initiation of chemotherapy for PTLD, the regimen was started for her. However, during the course of chemotherapy, she had a drop in peripheral blood oxygen saturation due to a seizure episode. Unfortunately, she ultimately died owing to complications from sepsis.

### Case 2

A 2-year-old Iranian (Persian) girl was referred to our center with fever, diarrhea, and bicytopenia. She had medical history of liver transplantation due to biliary atresia and had a known allergy to eggs. The patient did not have any positive family history of malignancy. Cervical lymph node enlargement was detected on physical examination, so an excisional biopsy was performed and diffuse infiltration of lymphoma cells positive for CD20 was reported. Further investigations, such as bone marrow aspiration, flow cytometry, and IHC, confirmed the diagnosis of PTLD (CD20, CD3, CD30, CD34, and PAX-5). Therefore, a proper chemotherapy regimen was started for her. However, 3 months later, she developed hematemesis and bloody diarrhea accompanied by the presence of multiple air–fluid levels on abdominal X-ray. Consequently, an emergency operation was performed, and multiple biopsies from the small intestine were taken that showed diffuse infiltration of atypical lymphocytes that were all CD20 positive. Unfortunately, she died owing to complications associated with PTLD.

### Case 3

A 2-year-old Iranian (Persian) boy was admitted to our hospital with prolonged intermittent fever for 3 months and several days of diarrhea prior to admission. He had medical history of cirrhosis due to biliary atresia and had undergone a liver transplantation about 16 months before being diagnosed with PTLD. Additionally, he had previous admissions for COVID-19 and multisystem inflammatory syndrome in children (MIS-C). There was no history of malignancy in the patient’s family. His recent tacrolimus level was recorded at 40 ng/ml. During the current admission, he developed bloody diarrhea, prompting the performance of a colonoscopy. The colonoscopy revealed two large volcano-like, clean base ulcers in the transverse and sigmoid colon. Biopsies were taken, and the pathology results demonstrated PTLD (CD20, CD3, Ki-67, and PAX-5 positive on IHC). A proper chemotherapy regimen was initiated for him. The timely diagnosis of PTLD was facilitated by the colonoscopy, enabling early intervention and management.

### Case 4

A 3.5-year-old Iranian (Persian) boy presented to our hospital with abdominal pain and diarrhea. He had medical history of liver transplantation due to progressive familial intrahepatic cholestasis (PFIC) 6 months before this admission. The patient did not have family history of malignancy. To investigate the cause of his chronic diarrhea, a stool sample was examined for different parasites, resulting in negative findings. Endoscopy showed several small and volcano-like ulcers in the duodenum. The pathology and immunohistochemistry testing of the intestinal biopsies confirmed high-grade monomorphic PTLD, B-cell type. Bone marrow aspiration and biopsy had no flow cytometry evidence of leukemia or lymphoma. A proper chemotherapy regimen was begun, but tragically he died owing to complications of PTLD and chemotherapy treatment after several weeks.

### Case 5

A 5-year-old Iranian (Persian) girl was referred to our hospital with right lower quadrant abdominal pain and rectal bleeding. She had liver transplantation due to Crigler–Najjar syndrome 5 months before this admission. She also had history of admission with abdominal pain and vomiting a month before PTLD diagnosis, in which sonography and abdominal X-ray were normal. However, she did not have a family history of malignancy. At that time, her tacrolimus serum level was reported at 27 ng/Ml, which led to a decrease in the tacrolimus dose. In this course of admission, despite normal findings on sonography, abdominal X-ray, and endoscopy and a decreased tacrolimus level of 20 ng/Ml, abdominal computed tomography (CT) scan revealed perforation. As a result, an emergency operation was performed during which five different perforation sites were found in the small bowel and biopsies were taken. Pathology and IHC confirmed PTLD diagnosis (positive for LCA, CD20, PAX-5, EBV, CMV, and CD79). She is currently receiving chemotherapy treatment (Additional file 1).

### Discussion and conclusion

PTLD is one of the most severe forms of posttransplant complication in children, and it induces significant morbidity and mortality in pediatric recipients of liver transplantation. According to literature, the incidence of PTLD is up to 15% in pediatric liver transplant recipients [7]. Despite a reduction in the incidence of PTLD in recent years, which might be due to preferred lower serum tacrolimus level, the increasing awareness of PTLD presenting signs and symptoms, and close monitoring of recipients’ EBV status [11–13], we observed an increase in the number of PTLD cases in our center in the last year. Besides, the average mortality rate of PTLD in pediatrics is 18–42% [6, 14]. However, three of our five patients died, which is higher than the expected rate.

This case series reported five cases with PTLD who presented with GI symptoms, which is an increase in number in comparison with previous studies at our center and reports from other centers. For example, one of the most extensive cross-sectional surveys conducted among patients who underwent liver transplantation at Shiraz Transplant Center between 2004 and 2015 reported 40 cases of PTLD in pediatrics with an incidence of 6.25%, which was comparable to other studies. Of these patients, 15 presented with abdominal pain and 5 of them with diarrhea [9]. In a retrospective cohort study of 98 pediatric liver transplant recipients at Johns Hopkins Hospital, 8 children had PTLD with a cumulative incidence of 8% in 20 years and 2 patients had gastrointestinal PTLD, and in 2 cases liver (graft) was the primary site of involvement [15]. In a 16-year single-center study in Turkey, including 236 patients who received liver transplant between 2001 and 2017, there were 8 cases of PTLD, and only 2 of them presented with diarrhea and abdominal pain [10].

The clinical features of PTLD are highly variable. The early symptoms can be nonspecific such as fever, malaise, and weight loss. Lymphadenopathy, chronic diarrhea, abdominal pain, and secondary presentations due to pressure effects such as gastrointestinal bleeding or bowel perforation with a surgical abdomen are among the practical manifestations. Fulminant PTLD is a rare condition characterized by disseminated systemic disease and septic shock [7, 8]. All of our patients presented with fever and gastrointestinal manifestations (e.g., diarrhea, abdominal pain). Despite our knowledge of PTLD clinical features, its initial nonspecific presentations can be misleading. The previously mentioned symptoms are frequently seen in immunosuppressive drug side effects or opportunistic viral infections such as viral gastroenteritis and COVID-19. Therefore, close follow-up of patients, monitoring of EBV status, awareness of subtle or overt symptoms of the disease, and taking precautions in the early phases can help physicians to screen recipients and improve survival [11, 13, 16, 17].

New EBV infection after transplantation or reactivation of previously acquired EBV was reported in the majority of PTLD cases. Immune response to EBV infection leads to uncontrolled B cell proliferation and tumor formation in a setting of immunosuppression. Patients with negative EBV tests before transplantation are at higher risk for acute primary EBV infection, especially in an immunosuppression background where the patient is predisposed to various viral infections such as primary cytomegalovirus (CMV) disease [6, 15]. It has been reported that the chance of developing PTLD is four to six times higher when there is a simultaneous CMV infection [15]. More evidence on COVID-19 and concomitant reactivation of EBV or CMV is required, although some studies have revealed that critically ill COVID-19 patients are at higher risk for EBV and CMV reactivation [20, 21]. Data on EBV and CMV infection in our cases are presented in Table 1.

As the number of PTLD cases in our center has increased over the last year during the COVID-19 pandemic, an essential question arises: Does this increase in the PTLD cases result from cancer care challenges during the COVID-19 pandemic (e.g., postponing treatment owing to fear of COVID-19 infection, limited healthcare resources during COVID-19 transmission peaks, social distancing, and lockdown policies), or is there a potential relationship between COVID-19 infection and reactivation of EBV and CMV? The fact that two of our patients had positive COVID-19 PCR tests before PTLD diagnosis and two of them had a COVID-19 record in their family members can support the latter hypothesis. This is a hypothesis that requires further research.

Inayat *et al.* recommended urgent endoscopy in patients with nonspecific GI manifestations after transplantation for early diagnosis of PTLD [18]. Furthermore, in a case study by Suzuki *et al.*, it was suggested that endoscopy could lead to early PTLD diagnosis [19]. All cases in this study had nonspecific GI symptoms. Early PTLD diagnosis was confirmed only after colonoscopy and endoscopy. Therefore, we recommend both colonoscopy and endoscopy in patients with nonspecific GI symptoms after liver transplantation. Early diagnosis with these procedures can prevent late presentations of PTLD, such as bowel perforation, and can improve the survival of such patients.

With this case series, we aim to raise awareness about fever, diarrhea, and abdominal pain as the first clinical manifestations of PTLD, even in symptomatic COVID-19 patients. In other words, the nonspecific symptomology of PTLD in COVID-19 patients should not lead to missing the PTLD diagnosis. Also, we formulated a hypothesis about the probable impact of COVID-19 on PTLD incidence and mortality. Finally, we recommend endoscopy and colonoscopy in patients with nonspecific GI symptoms after transplantation for early diagnosis of PTLD.

### Supplementary Information

**Additional file 1.** Multiple perforation of the small intestine of the fifth case caused by post-transplant lymphoproliferative disorder.

## Data Availability

Information regarding the patients can be requested from the authors. Do not hesitate to get in touch with the corresponding author if you are interested in such data.

## Abbreviations

PTLD: Posttransplant lymphoproliferative disorder
GI: Gastrointestinal
COVID-19: Coronavirus disease 2019
CMV: Cytomegalovirus
EBV: Epstein–Barr virus
PFIC: Progressive familial intrahepatic cholestasis
IHC: Immunohistochemistry
